# Editorial: Bridging the gap: implementing effective lifestyle interventions focusing on nutrition and physical activity for type 2 diabetes prevention in low and middle-income countries

**DOI:** 10.3389/fnut.2026.1932537

**Published:** 2026-09-16

**Authors:** Yingting Cao, Xiaoyue Xu, Sathish Thirunavukkarasu

**Affiliations:** 1 School of Allied Health, Human Services and Sport, La Trobe University, Melbourne, VIC, Australia; 2 Non-Communicable Diseases and Implementation Science, Baker Heart and Diabetes Institute, Melbourne, VIC, Australia; 3 School of Population Health, Faculty of Medicine and Health, University of New South Wales, Sydney, NSW, Australia; 4 Madras Diabetes Research Foundation, Chennai, India

**Keywords:** type 2 diabetes prevention, lifestyle factors, implementation gaps, evaluation, low and middle-income countries

Type 2 diabetes mellitus (T2D) continues to pose a major public health challenge worldwide, with low- and middle-income countries (LMICs) carrying an increasing share of the burden (1). Although landmark studies have demonstrated that lifestyle interventions focusing on healthy dietary behaviors, physical activity, and weight management can effectively prevent or delay the onset of T2D among high-risk populations (2, 3), translating these interventions into sustainable and scalable practice remains challenging, particularly in LMICs (4). Resource constraints, rapidly changing food environments, sociocultural influences, and health-system limitations all affect the implementation and long-term success of diabetes prevention efforts (5).

This Research Topic brings together eight articles that explore the challenges and opportunities in diabetes prevention and management across diverse settings. Collectively, these papers highlight not only what works for diabetes prevention, but also how evidence-based lifestyle interventions can be implemented, adapted and evaluated in real-world LMIC settings.

## Characterizing the diabetes challenge

Understanding the scale of the problem is a critical first step in designing effective prevention strategies. Huang et al. demonstrated that the global burden of T2D attributable to high body mass index (BMI) increased substantially from 1990 to 2021 and is projected to continue rising through 2045. The study highlighted important geographic, socioeconomic and gender disparities, with women experiencing a greater burden after around age 60. These findings provide a population-level rationale for prioritizing diabetes prevention and reinforce the importance of implementing effective lifestyle interventions that address the growing burden of high BMI and T2D across diverse settings.

## Understanding the structural, social, and behavioral drivers of diabetes prevention and management

A central theme emerging from this Research Topic is that knowledge alone is often insufficient to achieve meaningful and sustained behavior change. Instead, lifestyle behaviors are shaped by a complex interaction of structural, social, cultural, psychological, and behavioral factors.

McMillin et al. examined perceived barriers to fruit and vegetable intake and dietary self-efficacy among Indian adults at high risk of T2D. Their findings showed that barriers related to the availability and affordability of fruits and vegetables were more strongly associated with dietary intake than individual self-efficacy, highlighting the importance of addressing food-system constraints alongside behavior change strategies.

Gil et al. investigated diabetes self-care knowledge, attitudes, and practices among adults with T2D in Colombia. Although participants generally reported favorable knowledge, attitudes, and self-care practices, important gaps remained in dietary planning and blood glucose self-monitoring. These findings suggest that knowledge and positive attitudes alone may not be sufficient to translate into comprehensive diabetes self-management.

Rweyendera et al. emphasized the importance of patient support during the early stages of diabetes management. Among newly diagnosed individuals with T2D in Tanzania, participants demonstrated moderate diabetes knowledge, prevalent negative attitudes, and impaired quality of life, particularly in environmental and physical domains. These findings suggest that educational interventions alone may be insufficient, and highlighted the need to integrate emotional, social, and multidisciplinary support early in the disease trajectory.

Sanuade et al. presented a community-based qualitative study conducted in a low-income urban community in Accra, Ghana, involving older adults and key community stakeholders. Rather than focusing solely on individuals living with diabetes, the study explored community perceptions and contextual influences shaping diabetes prevention and management. Three key lessons emerged: first, knowledge alone may not be sufficient to translate into healthy behaviors due to structural constraints; second, food carries important cultural and social meanings beyond nutrition; and third, community members attributed diabetes to both lifestyle behaviors and broader environmental factors. These findings underscore the importance of culturally responsive, community-engaged approaches that address social and structural determinants alongside individual behavior change when designing lifestyle interventions in LMICs.

Behavioral complexity was further explored by Yu et al., who investigated night-eating behaviors among young and middle-aged adults with T2D in China. Applying the Fogg Behavior Model, the authors identified four distinct behavioral subtypes influenced by physiological stress, emotional and psychological factors, social environments, and health-related cognitive biases. The study demonstrated that similar dietary behaviors may arise from very different motivations and contextual triggers, supporting the need for more personalized intervention strategies.

Although conducted across different settings and populations, these studies convey a common message: successful implementation requires more than simply increasing awareness and knowledge. Sustainable behavior change depends on addressing structural barriers, understanding social and cultural contexts, recognizing individual experiences, and tailoring interventions to diverse behavioral needs.

## Strengthening implementation science through improved evaluation

Beyond understanding behavior and context, effective implementation requires robust tools for assessment and evaluation. Two studies in this Research Topic contribute directly to strengthening implementation and evaluation of lifestyle interventions in LMICs.

Zhang et al. developed and validated a diabetes-specific nutrition literacy scale for individuals with T2D in China. Comprising four dimensions and 31 items, the scale demonstrated good reliability and validity and addresses an important measurement gap. The tool may support more targeted educational interventions and evaluation of nutrition-related programs in Chinese populations and potentially inform adaptation in other settings.

Daivadanam et al. conducted a global consensus process to identify and prioritize a core set of disease-related measures for use in diabetes research. By establishing agreement on what should be measured and reported across studies and contexts, the authors addressed a longstanding challenge in diabetes research and provided practical guidance for improving consistency in outcome assessment and evaluation.

Together, these studies strengthen the implementation infrastructure for diabetes prevention by improving both the measurement of nutrition-related determinants and the standardization of core disease-related measures.

## Concluding remarks

Collectively, the studies in this Research Topic demonstrate that translating effective lifestyle interventions into routine practice requires an integrated understanding of disease burden, behavioral determinants, sociocultural context and robust evaluation frameworks.

Several implications have emerged across this Research Topic. First, the rising burden of T2D attributable to high BMI underscores the need to strengthen, adapt and scale evidence-informed prevention strategies that are responsive to local contexts. Second, lifestyle interventions must go beyond improving knowledge alone and address the structural, social, cultural, and psychological conditions that enable or constrain sustained behavior change. Finally, stronger and more standardized measurement approaches are needed to evaluate intervention delivery, compare findings across settings, and guide future diabetes prevention research and practice.

These insights provide a valuable foundation for future implementation research and for policies and programs aimed at reducing the growing global burden of T2D.
